# *Lactobacillus reuteri* 6475 Increases Bone Density in Intact Females Only under an Inflammatory Setting

**DOI:** 10.1371/journal.pone.0153180

**Published:** 2016-04-08

**Authors:** Fraser L. Collins, Regina Irwin, Hayley Bierhalter, Jonathan Schepper, Robert A. Britton, Narayanan Parameswaran, Laura R. McCabe

**Affiliations:** 1 Department of Physiology, Michigan State University, East Lansing, Michigan, United States of America; 2 Department of Molecular Virology and Microbiology, Baylor College of Medicine, Houston, Texas, United States of America; 3 Department of Radiology, Michigan State University, East Lansing, Michigan, United States of America; 4 Biomedical Imaging Research Centre, Michigan State University, East Lansing, Michigan, United States of America; University of Massachusetts Medical, UNITED STATES

## Abstract

**Background & Aims:**

We previously demonstrated that short-term oral administration of the probiotic *Lactobacillus reuteri* 6475 enhanced bone density in male but not female mice. We also established that *L*. *reuteri* 6475 enhanced bone health and prevented bone loss in estrogen-deficient female mice. In this study, we tested whether a mild inflammatory state and/or a long-term treatment with the probiotic was required to promote a positive bone effect in estrogen-sufficient female mice.

**Methods:**

A mild inflammatory state was induced in female mice by dorsal surgical incision (DSI). Following DSI animals were orally supplemented with *L*. *reuteri* or vehicle control for a period of 8 weeks. Gene expression was measured in the intestine and bone marrow by qPCR. Distal femoral bone density and architecture was analyzed by micro-CT.

**Results:**

We report that 8 weeks after DSI there is a significant increase in the weight of spleen, thymus and visceral (retroperitoneal) fat pads. Expression of intestinal cytokines and tight junction proteins are also altered 8 weeks post-DSI. Interestingly, *L*. *reuteri* treatment was found to display both intestinal region- and inflammation-dependent effects. Unexpectedly we identified that 1) *L*. *reuteri* treatment increased bone density in females but only in those that underwent DSI and 2) DSI benefited cortical bone parameters. In the bone marrow, dorsal surgery induced CD4^+^ T cell numbers, a response that was unaffected by *L*. *reuteri* treatment, whereas expression of RANKL, OPG and IL-10 were significantly affected by *L*. *reuteri* treatment.

**Conclusion:**

Our data reveals a previously unappreciated effect of a mild surgical procedure causing a long-lasting effect on inflammatory gene expression in the gut and the bone. Additionally, we demonstrate that in intact female mice, the beneficial effect of *L*. *reuteri* on bone requires an elevated inflammatory status.

## Introduction

Throughout life the adult human skeleton is continuously remodeled with approximately 5–10% of the existing bone replaced every year [1]. Remodeling is accomplished through the coupled activities of osteoclasts, cells responsible for the degradation of bone, and osteoblasts, cells that produce organic bone matrix and promote its mineralization [2,3]. The balanced process of bone remodeling is negatively influenced by numerous factors. Up-regulated expression of pro-inflammatory and pro-osteoclastogenic cytokines, such as tumor necrosis factor (TNF), interleukin (IL)-1β, interferon gamma (IFNγ) and receptor activator of nuclear factor-kappa B ligand (RANKL), have been shown to disrupt bone remodeling and lead to bone loss [4–7]. In contrast, increased levels of the anti-osteoclastogenic cytokine osteoprotegerin (OPG) or the anti-inflammatory cytokine IL-10 increase bone density by directly inhibiting osteoclast formation or inhibiting production of pro-inflammatory cytokines [8–10]. Consistent with a role for local inflammation in bone density regulation, rheumatoid arthritis (RA), periodontitis, loosened joint prostheses and tooth implants, all increase inflammation in close proximity to bone and can cause local increases in RANKL and pro-inflammatory cytokines, leading to bone loss [11]. In addition, chronic diseases associated with systemic inflammation (marked by elevated serum cytokine levels) can also promote bone loss that is distant from the site of initiation of inflammation as seen in periodontitis; in autoimmune diseases such as psoriasis, type 1 diabetes, RA and inflammatory bowel disease (IBD) [11–14]; as well as in aging [15].

In recent years, the intestinal microbiota has emerged as a potentially important regulator of systemic health. Dysbiosis of the intestinal microbiota is linked to the pathogenesis of a number of diseases including diabetes, obesity, IBD, RA and liver disease which are also associated with adverse bone pathology [16–22]. Studies have demonstrated that modulation of the microbiota with probiotic bacteria is able to reduce disease processes and a few studies, including studies from our lab, further establish that probiotics can prevent bone pathology complications [23–25]. Additionally, our studies have also highlighted the beneficial effect of probiotic supplementation on general bone health under non-diseased conditions [26]. In these studies, we found that the effect of *L*. *reuteri* on bone is gender-dependent. In particular, we demonstrated that 4-week supplementation with *L*. *reuteri* 6475 increased bone density in male but not female mice [26]. We later demonstrated that estrogen deficient female mice (ovariectomized model) respond to oral *L*. *reuteri* 6475 administration and display bone health benefits. Specifically, treatment with probiotic bacteria protected against estrogen deficiency-induced trabecular and cortical bone loss [23,24]. Given that estrogen is known to be an important immunomodulator, it was not entirely surprising that some aspects of immune components were altered in the ovariectomized mice. Immune responses have previously been shown to differ between males and females. For example, in models of sepsis females with *H*. *hepaticus*-induced colitis and lipopolysaccharide (LPS)-induced airway inflammation display reduced immune response and/or increased survival compared to males [27–29]. Taken together, this suggests that estrogen can lower baseline inflammatory status and modulate immune responses to insult.

The studies noted above made us question if a minor surgical procedure could induce inflammation and affect responses to *L*. *reuteri* treatment, especially under estrogen sufficient conditions in females. We utilized dorsal surgical incision (DSI), a procedure commonly used in ovariectomy and sham mouse surgery studies that represents a precise, reproducible cut in the skin. Remarkably, we report that DSI in female mice induces several inflammatory components observed as long as 8 weeks after the procedure. Interestingly, while *L*. *reuteri* has no effect on intact female mice (consistent with previous observations [26]), it increased femoral trabecular bone density in those that had undergone DSI. Our findings suggest that in the presence of estrogen, a mild inflammatory status is required for *L*. *reuteri* to exert a beneficial effect on the bone.

## Materials and Methods

### Animals and Experimental Design

Female Balb/c mice 11 weeks of age were obtained from The Jackson Laboratory (Bar Harbour, Maine). Mice were allowed to acclimate to animal facility for 1 week prior to start of experiment. After 1 week mice were randomly split into four groups (9–18 per group): control (+/- probiotic) or dorsal surgical incision (+/- probiotic). DSI entailed placing mice under isofluorane anesthesia for < 5 minutes and making a 2 cm lower-mid dorsal incision extending through the skin and muscle layer and then using surgical staples to close the site. Two days after surgery mice were treated by gavaging with 300 μl (1x10^9^ cfu/ml) *L*. *reuteri* 6475 or MRS broth (vehicle control), three times per week for 4 or 8 weeks. *L*. *reuteri* was additionally added to the drinking water (of *L*. *reuteri* treated groups only) at a concentration of 3.3x10^8^ cfu/ml. Mice were given Teklad 2019 chow (Madison, WI) and water ad libitum and were maintained on a 12 h light/dark cycle. All animal procedures were approved by the Michigan State University Institutional Animal Care and Use Committee and conformed to NIH guidelines.

### Bacterial Culture Conditions

*L*. *reuteri* ATCC PTA 6475 was initially cultured on deMan, Rogosa, Sharpe media (MRS, Difco)—agar plates and kept under anaerobic conditions at 37°C for a maximum of 1 week. For gavaging of all mice, one colony of *L*. *reuteri* was picked and anaerobically cultured in 10ml of MRS broth for 16-18h at 37°C. MRS broth was additionally used as a negative control. For drinking water, *L*. *reuteri* was anaerobically cultured in 10 ml of MRS broth for 16-18h at 37°C. The following day, the overnight culture was sub-cultured into fresh MRS and grown until log phase (OD_600_ = 0.4). *L*. *reuteri* was spun down, re-suspended in sterile PBS, cfu/ml calculated and stored at -80°C until use. *L*. *reuteri* was re-suspended in drinking water at a final concentration of 3.3x10^8^ cfu/ml.

### μCT Bone Imaging

Fixed femurs were scanned using a GE Explore Locus microcomputed tomography (μCT) system at a voxel resolution of 20μm obtained from 720 views. Beam angle of increment was 0.5, and beam strength was set at 80 peak kV and 450 uA. Each run consisted of control (non-surgery and DSI) and *L*. *reuteri*- treated mouse bones, and a calibration phantom to standardize grayscale values and maintain consistency. Bone measurements were performed blind. Femoral bone analyses were performed in a region of trabecular bone defined at 1% of the total length proximal to the growth plate and extending 2 mm toward the diaphysis excluding the outer cortical bone. Trabecular bone mineral content, bone volume fraction, thickness, spacing, and number values were computed by a GE Healthcare MicroView software application for visualization and analysis of volumetric image data. Cortical measurements were performed in a 2x2x2 mm cube centered midway down the length of the bone.

### Femoral Dynamic Measures

For dynamic histomorphometric measures of bone formation, mice were injected intraperitoneally with 200 μl of 10 mg/ml calcein (Sigma, St. Louis, MO) dissolved in sterile saline at 7 and 2 days prior to harvest. Femora (n = 8–12 per group) were embedded in paraffin blocks sectioned, viewed under a fluorescent microscope and digital images obtained. The distance between the calcein lines (mineral apposition rate, MAR) and their length along the bone surface were measured and used to calculate bone formation rate (BFR).

### Flow Cytometric Analysis

Following euthanasia femora were cleaned of muscle and bone marrow (BM) cells isolated by either flushing or spinning (n = 10–18 per group). 1x10^6^ cells were incubated with Fc block (BD Pharmingen, CA, USA) for 15 min. Cells were stained with anti-mouse CD3-AlexaFluor 700 (500A2, eBioscience), anti-mouse CD4-FITC (RM 4–5, eBioscience) and anti-mouse CD8a-PE-Cyanine5.5 (5–6.7, eBioscience) for 30 minutes before fixing in formaldehyde. Data were acquired on a BD LSRII (Becton Dickinson, Franklin Lakes, NJ) and analyzed with FlowJo (Version 10; FlowJo, LLC, Ashland OR).

### Bone Marrow and Intestine RNA Analysis

Bone marrow was obtained as described above. Immediately following euthanasia, intestines were cleaned of connective tissue and luminal contents, snap frozen in liquid nitrogen and stored at -80°C. Frozen intestines were crushed under liquid nitrogen conditions with a Bessman Tissue Pulverizer (Spectrum Laboratories, Rancho Dominguez, CA). RNA was isolated from frozen samples (n = 6–10 per group) using TriReagent (Molecular Research Center, Cincinnati, OH) and integrity assessed by formaldehyde-agarose gel electrophoresis. cDNA was synthesized by reverse transcription using Superscript II Reverse Transcriptase Kit and oligo dT(12–18) primers (Invitrogen, Carlsbad, CA). cDNA was amplified by quantitative PCR (qPCR) with iQ SYBR Green Supermix (BioRad, Hercules, CA), and gene specific primers (synthesized by Integrated DNA Technologies, Coralville, IA; Table 1). Hypoxanthine guanine phosphoribosyl transferase (HPRT) mRNA levels were used as an internal control.

Real time PCR was carried out for 40 cycles using the iCycler (Bio-Rad) and data evaluated using the iCycler software. Each cycle consisted of 95°C for 15 sec, 60°C for 30 sec and 72°C for 30 sec. Negative controls included primers without cDNA.

**Table 1:** qPCR Primers.

Gene	Forward (5’-3’)	Reverse (5’-3’)
HPRT	AAGCCTAAGATG AGCGCAAG	TTACTAGGCAGATGGCCACA
OPG	TGGAGATCGAATTCTGCTTG	TCAAGTGCTTGAGGGCATAC
RANKL	TTTGCAGGACTCGACTCTGGAG	TCCCTCCTTTCATCAGGTTATGAG
IL-10	GGTTGCCAAGCCTTATCGGA	ACCTGCTCCACTGCCTTGCT
TGFβ1	GCAACAATTCCTGGCGTTACC	CCCTGTATTCCGTCTCCTTGGT
TNF	AAGGGAGAGTGGTCAGGTTGCC	CCTCAGGGAAGAGTCTGGAAAGG
IFNγ	GGCTGTCCCTGAAAGAAAGC	GAGCGAGTTATTTGTCATTCGG
IL-1β	TCCCCGTCCCTATCGACAAAC	GCGGTGATGTGGCATTTTCTG
Occludin	GCTCAGGGAATATCCACCTAT	CACAAAGTTTTAACTTCCCAGACG

### Serum Measurements

Blood was collected at the time of harvest, allowed to clot at room temperature for 5 min, then centrifuged at 5000g for 10 min. Serum was removed, aliquoted and frozen in liquid nitrogen, and stored at -80°C. Serum tartrate resistant acid phosphatase 5b (TRAP5b) and Osteocalcin (OC) were measured using Mouse TRAP (SB-TR103, Immunodiagnostic Systems Inc., Fountain Hills, AZ) and OC assay kits (BT-470, Biomedical Technologies Inc., Stoughton, MA), respectively, according to the manufacturer’s protocol (n = 7–18 per group).

### Statistical Analysis

All measurements are presented as the mean ± SEM. Significant outliers were removed using the Grubb’s test for outliers. 1-way ANOVA was performed using GraphPad Prism software version 6 (GraphPad, San Diego, CA, USA). A *p*-value ≤0.05 was considered significant.

## Results

### Effect of Dorsal Surgical Incision (DSI) and *L*. *reuteri* on General Body Parameters

**Fig 1:**
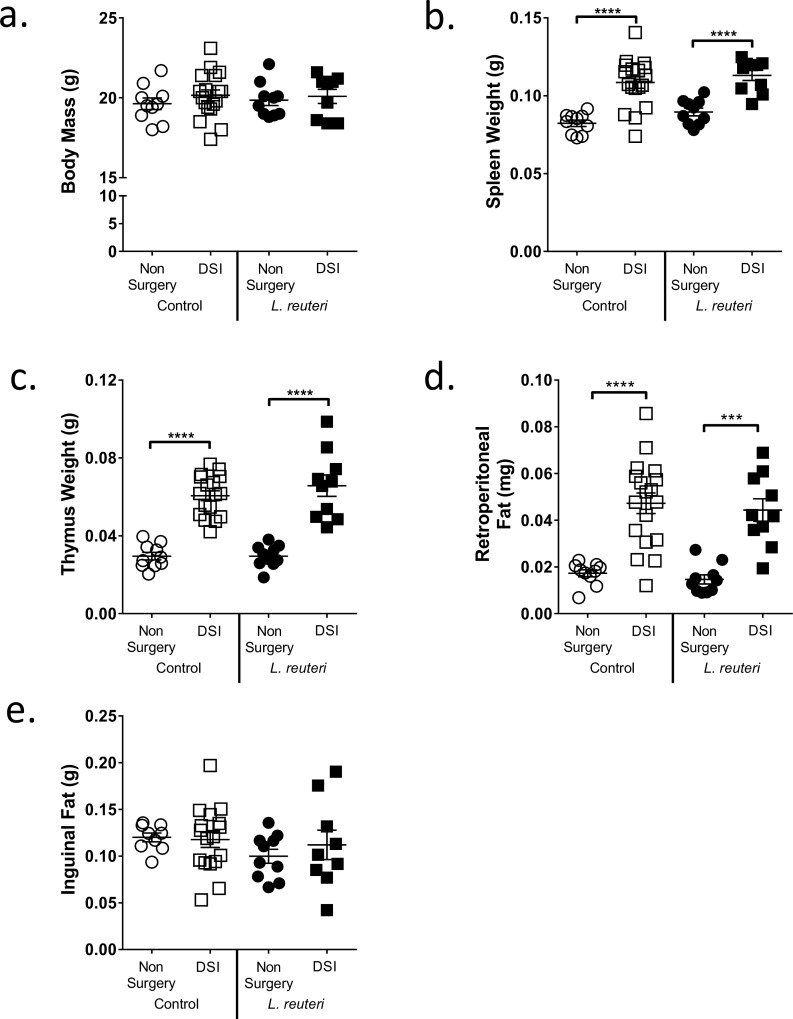
DSI Increases General Markers of Inflammation. a) Body Mass, b) spleen c) thymus d) retroperitoneal fat and e) inguinal fat were weighed after an 8 week period in non-surgery and DSI mice ± *L*. *reuteri*. DSI significantly increased spleen weight (*p*<0.0001), thymus weight (*p*<0.0001) and retroperitoneal fat (*p*<0.001) but had no significant effect on body mass or inguinal fat. n = 9–18 per group. Data is mean ± SEM. Statistical analysis performed by 1-way ANOVA with Fisher’s LSD post-test.

Previous studies demonstrated that supplementation with *L*. *reuteri* for 4 weeks significantly increased femoral bone density in specific-pathogen free male mice and prevented ovariectomy-induced bone loss in female mice. However, 4 weeks of *L*. *reuteri* treatment failed to have a significant effect on bone density in intact female mice [23,26]. Therefore, we investigated whether long-term (8 weeks) *L*. *reuteri* supplementation could modulate bone density in intact female mice and also tested whether an underlying inflammatory condition was required to obtain a beneficial bone response. Intact mice and mice that underwent DSI were given either *L*. *reuteri* 6475 or MRS broth (vehicle) orally by drinking water and by gavage 3x a week for the duration of the study. Mice were sacrificed at the end of the study and general body parameters measured (Fig 1). Interestingly, DSI mice (± *L*. *reuteri*) displayed significantly increased weight of spleen (*p*<0.001), thymus (*p*<0.001) and retroperitoneal fat (*p*<0.01) when compared to non-surgery mice 8 weeks post-surgery. DSI and *L*. *reuteri* had no significant effects on body mass and inguinal fat mass compared to the respective controls.

### Assessing the Impact of DSI and *L*. *reuteri* on Trabecular Bone Volume Fraction and Cortical Bone

**Fig 2:**
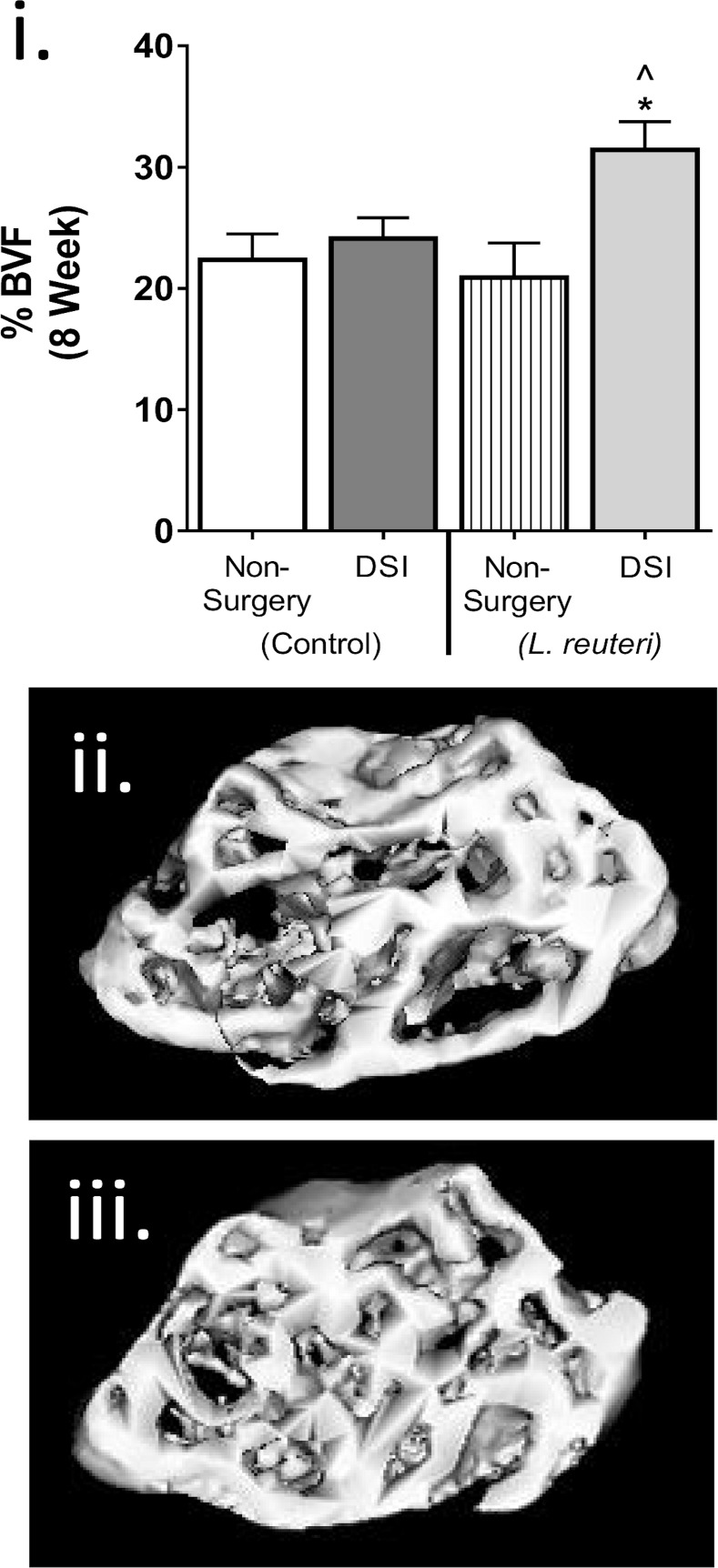
*L*. *reuteri* Significantly Increases Bone Volume in Female Mice 8 Weeks after DSI. Non-surgery and DSI mice were treated ± *L*. *reuteri* for 8 weeks and trabecular bone density analyzed by μCT. i) *L*. *reuteri* significantly increased %BVF in the DSI mice compared to DSI control (*p*<0.05) and non-surgery control (*p*<0.05). No significant difference was observed between the non-surgery and DSI controls. *L*. *reuteri* had no effect in the non-surgery cohort. Representative μCT isosurface images of ii) DSI and iii) DSI + *L*. *reuteri*. n = 8–18 per group. Statistical analysis performed by 1-way ANOVA with Fisher’s LSD post-test.

To assess whether long-term *L*. *reuteri* supplementation benefits bone health, we examined the distal femur metaphyseal trabecular region by microcomputed tomography 8 weeks after the start of treatment (Table 2). Remarkably, we identified a significant increase (nearly 50%) in bone volume fraction (BVF; Fig 2) in the DSI + *L*. *reuteri* cohort compared to the DSI + broth group (*p*<0.05) and non-surgery control (*p*<0.05). Consistent with this finding, trabecular number (Tb. N, *p*<0.05) increased and trabecular spacing (Tb. Sp.) decreased (*p*<0.05) in the *L*. *reuteri* treated DSI cohort compared to the non-surgery control (Table 2). In contrast, we did not observe any change in BVF in the non-surgery + *L*. *reuteri* mice compared to the non-surgery controls, consistent with previous observations [26]. Analysis of femoral diaphysis cortical parameters (Table 2) revealed that DSI had a major effect on the cortical bone structure and strength. Specifically, DSI significantly increased inner perimeter (*p*<0.01), outer perimeter (*p*<0.01), marrow area (*p*<0.05), cortical area (*p*<0.05), total area (*p*<0.0001), bone mineral content (*p*<0.05) and moment of inertia (*p*<0.01) when compared to the non-surgery control. *L*. *reuteri* had no effect on cortical parameters in either the non-surgery or DSI treated cohorts.

**Table 2:** Femoral and Cortical Bone Parameters.

**Femur Trabecular Bone Parameters**	Non-Surgery	DSI	Non-Surgery+ *L*. *reuteri*	DSI+ *L*. *reuteri*
% BVF	22.4±2.1	24.1±1.7	20.9±2.8	31.4±2.3*
Tb. N. (1/mm)	4.44±0.31	5.18±0.23	4.26±.030	5.63±0.37*
Tb. Th. (μm)	48±2	45±2	47±3	50±2
Tb. Sp. (mm)	0.19±0.01	0.15±0.01	0.20±0.02	0.13±0.01*
BFR(μm)	1.80±0.31	2.82±0.36	2.03±0.50	2.95±0.44
MAR (μm/day)	0.26±0.02	0.50±0.09*	0.39±0.10	0.62±0.16*
**Femoral Cortical Bone Parameters**	Non-Surgery	DSI	Non-Surgery+ *L*. *reuteri*	DSI+ *L*. *reuteri*
Inner Perimeter (mm)	2.65±0.04	2.81±0.04**	2.71±0.02	2.83±0.04**
Outer Perimeter (mm)	4.22±0.05	4.46±0.04**	4.29±0.03	4.54±0.05**
Marrow Area (mm^2^)	0.48±0.01	0.55±0.02**	0.51±0.01	0.56±0.02
Cortical Area (mm^2^)	0.84±0.02	0.93±0.02**	0.87±0.01	0.98±0.03**
Total Area (mm^2^)	1.32±0.03	1.48±0.02**	1.38±0.02	1.54±0.03**
BMD (mg/cc)	857±17	905±32	844±10	821±32
BMC (μg)	14.5±0.5	16.9±0.5**	14.7±0.3	16.2±0.8*
MOI (mm^4^)	0.24±0.01	0.30±0.01**	0.26±0.01	0.33±0.01**

Femur distal trabecular bone parameters. % BVF, bone volume fraction; Tb. N., trabecular number; Tb. Th., trabecular thickness; Tb. Sp., trabecular spacing; MAR, mineral apposition rate; BFR, bone formation rate; MOI, the cross sectional moment of inertia at the z-axis. Values are averages ± SEM. N = 7–18 per group. * Significant compared to non-surgery control

* *p*<0.05

** *p*<0.01. Statistical analysis performed with 1 Way ANOVA followed by or Fisher’s LSD post-test.

### Long-term Effects of DSI and *L*. *reuteri* on Intestinal Cytokine Gene Expression

**Fig 3:**
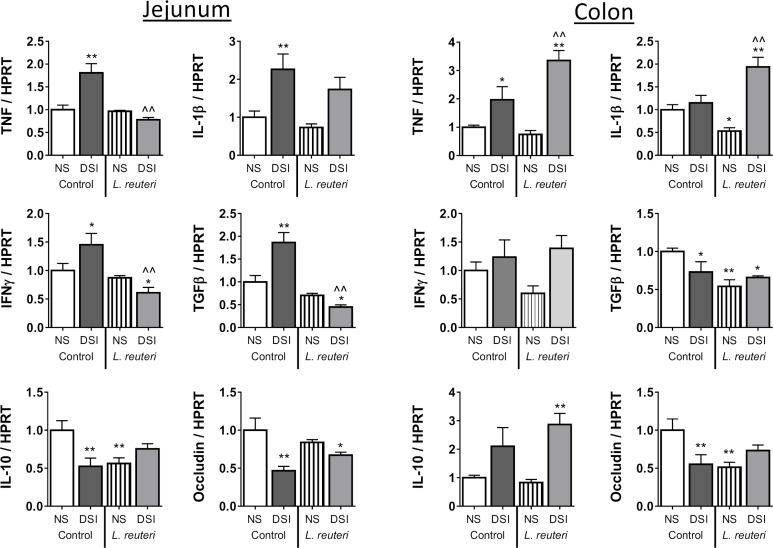
DSI and *L*. *reuteri* Significantly Alter Regional Intestinal Gene Expression. Non-surgery and DSI mice were treated ± *L*. *reuteri* for 8 weeks and gene expression analyzed in the jejunum and colon by qPCR. In the jejunum DSI significantly increased expression of TNFα (*p*<0.001), IL-1β (*p*<0.01), IFNγ (*p*<0.05) and TGFβ (*p*<0.001). Significant decreases in IL-10 (*p*<0.01) and occludin (*p*<0.01) were observed. In the non-surgery cohort *L*. *reuteri* significantly reduced IL-10 (*p*<0.01) expression. In the DSI cohort significant reductions were observed for TNFα (*p*<0.0001), IFNγ (*p*<0.001) and TGFβ (*p*<0.0001). In the colon, DSI resulted in a significant increase in TNFα (*p*<0.05) and a decrease in occludin expression (*p*<0.05). *L*. *reuteri* significantly reduced expression of occludin (*p*<0.05), IL-1β (*p*<0.05) and TGFβ (*p*<0.01) in the non-surgery cohort. In the DSI cohort, *L*. *reuteri* significantly increased expression of TNFα (*p*<0.01) and IL-1β (*p*<0.01) while increasing expression of IL-10. n = 8–10 per group. * = significant to non-surgery control, ^ = significant to DSI control. Statistical analysis performed by 1-way ANOVA with Fisher’s LSD post-test.

Knowing that DSI has systemic effects on bone, we wondered if DSI also affected the GI tract, contributing to the bone changes induced by surgery and *L*. *reuteri* supplementation. To test this hypothesis, we examined genes associated with gut-bone signaling pathways: jejunal and colonic gene expression of markers of inflammation (pro- and anti-inflammatory cytokines) and genes involved in gut barrier function (occludin) (Fig 3) which have been suggested to be involved in bone density regulation [30].

In the jejunum, DSI promoted inflammation as indicated by significant increases in gene expression of TNFα (*p*<0.001), IL-1β (*p*<0.01), TGFβ (*p*<0.001), IFNγ (*p*<0.05) and significantly decreased expression of IL-10 (*p*<0.01) and occludin (*p*<0.01). This was consistent with our hypothesis of DSI altering gut homeostasis, even 8 weeks after surgery. Even more interesting, some of these changes induced by DSI were reversed by *L*. *reuteri* treatment: expression of TNFα (*p*<0.0001), IFNγ (*p*<0.001) and TGFβ (*p*<0.0001) were decreased while occludin expression was increased in *L*. *reuteri* treated DSI group. Supplementation of the non-surgery cohort with *L*. *reuteri* significantly decreased expression of IL-10 (*p*<0.01). This was interesting even though intact females do not exhibit any bone effect following *L*. *reuteri* supplementation.

Compared to the jejunum, colonic TNFα significantly increased (*p*<0.05) while IL-10 trended upwards 8 weeks post-DSI. As in the jejunum, expression of occludin (*p*<0.01), and TGFβ (*p*<0.05) were significantly decreased in DSI mice. Unexpectedly, *L*. *reuteri* supplementation of the DSI group caused significant increases in expression of TNFα (*p*<0.01, 4-fold), IL-1β (*p*<0.01, 4-fold) and increased IL-10 (3-fold) compared to treated non-surgery mice. These increases were much greater than what was seen in the jejunum. In contrast, and more consistent with previous studies, *L*. *reuteri* treatment of intact female mice led to a significant decrease in occludin (*p*<0.01), IL-1β (*p*<0.05) and TGFβ (*p*<0.01) expression in the non-surgery cohort. Together, these results suggest that *L*. *reuteri* differentially affects intestinal gene expression depending on the intestinal segment and on presence or absence of a distant surgical stress.

### Modulation of the Bone Marrow CD4^+^ T Cell Population by DSI and *L*. *reuteri*

**Fig 4:**
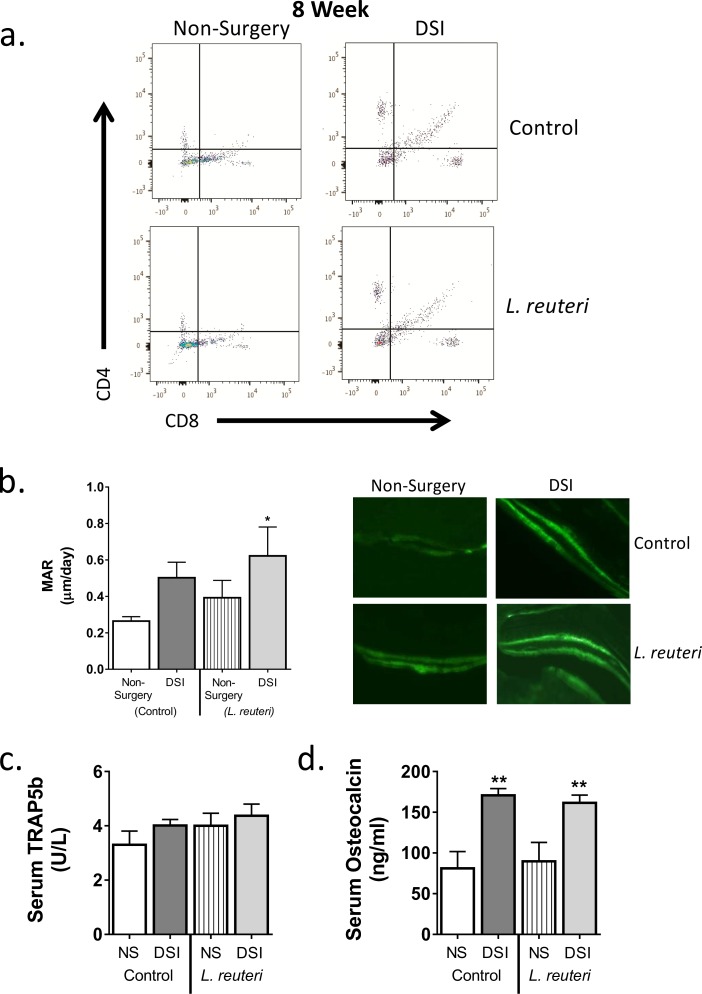
DSI Increases CD4^+^ T Cell Numbers in Bone Marrow and Significantly Increases Markers of Osteoblast Bone Formation after 8 Weeks. Non-surgery and DSI mice were treated ± *L*. *reuteri* for 8 weeks. Bone marrow was isolated and CD4^+^ T cell numbers analyzed by flow cytometry and osteoclast and osteoblast bone remodeling markers analyzed. a) Representative flow cytometry scatter plots. CD4^+^ T cells numbers were significantly increased in DSI mice (*p*<0.0001) compared to the non-surgery cohort. *L*. *reuteri* had no significant effect on CD4^+^ T cell numbers. b) MAR was measured by calcein incorporation, DSI + *L*. *reuteri* significantly increased MAR compared to non-surgery controls (*p*<0.05). Representative fluorescent images of calcein incorporation are included on the right. c, d) DSI had no effect on serum TRAP5b (c) but significantly increased levels of serum osteocalcin (d) compared to the non-surgery control (*p*<0.01). *L*. *reuteri* had no effect on TRAP5b or osteocalcin levels. n = 7–18 per group. * = significant to non-surgery control, ^ = significant to DSI control. Data is mean ± SEM. Statistical analysis performed by 1-way ANOVA with Tukey post-test or Fisher’s LSD test.

To determine whether DSI and/or oral *L*. *reuteri* administration could lead to a bone marrow (BM) cellular composition that is associated with changes in bone density, we analyzed BM CD4^+^ T cell numbers by flow cytometry at 8 weeks (Fig 4). BM CD4^+^ T cells have been shown to be important regulators of bone health [31]. Eight weeks following DSI, we identified a significant increase in the percentage of BM CD4^+^ T cells in the DSI group (17.7±1.1%) compared to the non-surgery cohort (6.8±0.7%; *p*<0.0001). *L*. *reuteri* treatment had no effect on CD4^+^ T cell numbers. No significant difference was observed in CD8^+^ T cell numbers between any of the conditions. These results suggest that the *L*. *reuteri* effect on bone density in the DSI group is likely independent of CD4^+^ T cell changes in the BM.

### Analysis of Osteoblast/Osteoclast Bone Remodeling Markers

**Fig 5:**
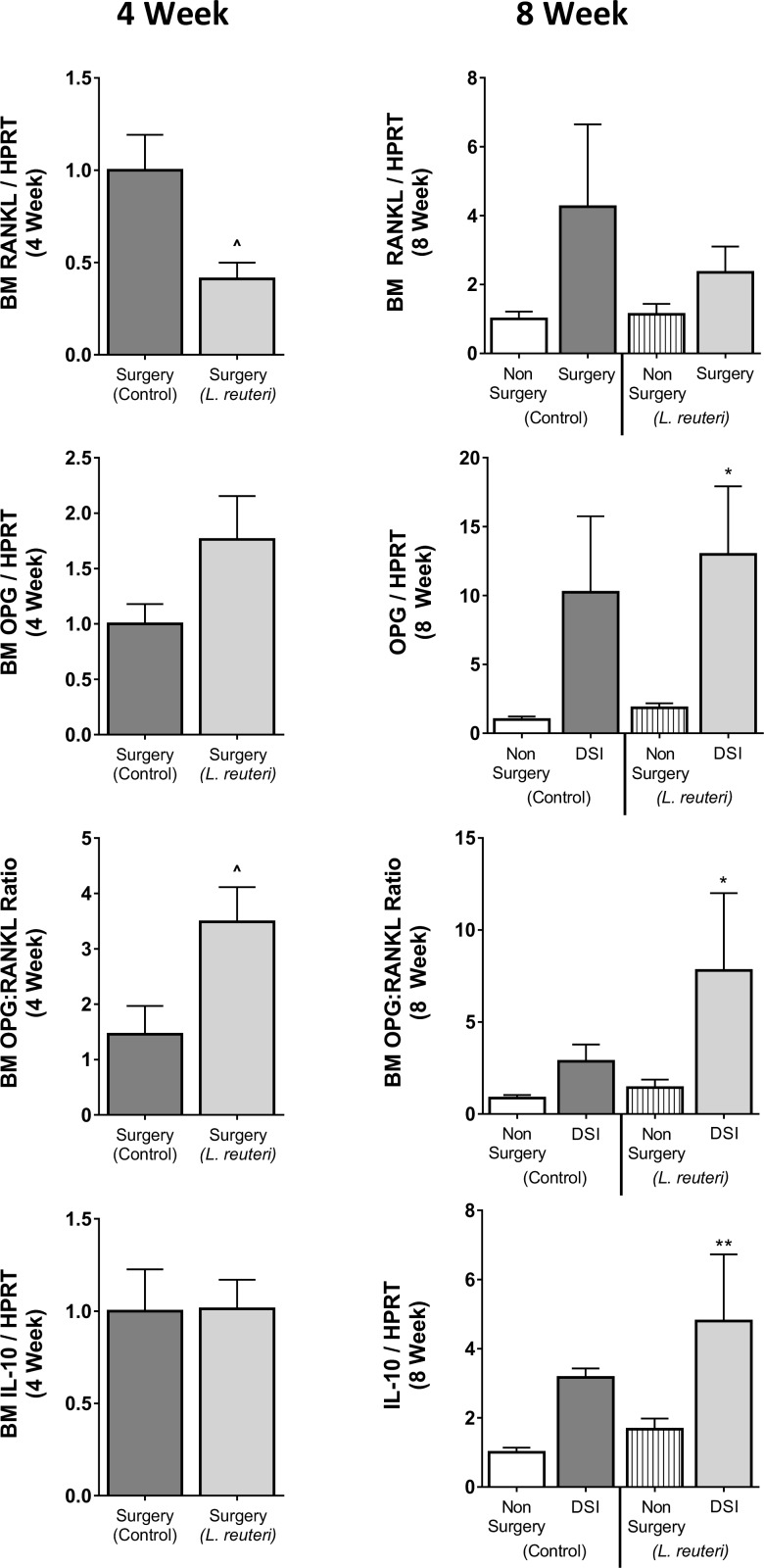
DSI and *L*. *reuteri* Alter Bone Marrow Gene Expression at 4 and 8 Weeks. Bone marrow was isolated from 4 and 8 week Non-surgery and DSI mice ± *L*. *reuteri* and gene expression analyzed by qPCR. At 4 weeks *L*. *reuteri* significantly reduced RANKL expression in the DSI cohort (*p*<0.05). *L*. *reuteri* additionally increased OPG expression resulting in a significant increase in the OPG:RANKL ratio (*p*<0.05) compared to the DSI control. At 8 weeks RANKL and OPG expression trended higher in the DSI control compared to the non-surgery control, resulting in an increased OPG:RANKL ratio. IL-10 expression was elevated in the DSI control over the non-surgery control. In the non-surgery cohort *L*. *reuteri* increased OPG and IL-10 expression. In the DSI cohort, *L*. *reuteri* decreased RANKL while increasing OPG expression resulting in a trend towards an increased OPG:RANKL ratio. n = 6–10 per group. * = significant to non-surgery control, ^ = significant to DSI control. Statistical analysis performed by 1-way ANOVA with Fisher’s LSD test.

To determine whether *L*. *reuteri* supplementation decreased catabolic and/or increased anabolic bone responses, markers of osteoclast and osteoblast activity were measured in bone, BM and serum at 8 weeks. Serum levels of osteocalcin were significantly increased in the DSI cohort (2.1-fold-170.7 ± 8.5 ng/ml, *p*<0.01) compared to the non-surgery controls (81.1 ± 20.6 ng/ml; Fig 5). TRAP5b levels, a specific marker of osteoclast activity, were modestly increased in the DSI group (4.0 ± 0.2 U/L) compared to non-surgery controls (3.3 ± 0.5 U/L). *L*. *reuteri* treatment had no significant effect on serum TRAP5b and osteocalcin levels in either the non-surgery or DSI groups. Consistent with the serum osteocalcin levels, analysis of calcein incorporation, a dynamic measure of bone formation, identified an increase in mineral apposition rate in the DSI mice (0.50 ± 0.09μm/day) and DSI + *L*. *reuteri* mice (0.62 ± 0.16μm/day; *p*<0.05) compared to the non-surgery controls (0.26 ± 0.02 μm/day) (Fig 4). Consistent with the changes in MAR, BFR trended to be higher in the DSI cohorts compared to the non-surgery controls, though this increase was not significant (Table 2). To further assess the effect of DSI and/or *L*. *reuteri* on BM, we examined the expression of the osteoclastogenic cytokine RANKL, the anti-osteoclastogenic cytokine OPG and the anti-inflammatory cytokine IL-10 (Fig 5). Eight weeks post-surgery, expression of RANKL and OPG were increased in the DSI group compared to the non-surgery group (4.3- and 10.2-fold respectively). This resulted in an increase in the OPG:RANKL ratio in the DSI control group compared to the non-surgery controls. In addition, IL-10 expression was enhanced in the DSI controls compared to the non-surgery controls. None of these parameters were affected significantly by *L*. *reuteri* treatment in the DSI group. Because of the observed bone effects at 8-weeks post-surgery/treatment, we reasoned that gene expression changes may have occurred earlier in the process. Therefore, we examined expression of the above-described genes in the BM from mice that underwent DSI ± *L*. *reuteri* 4 weeks after surgery. As posited, at 4 weeks, *L*. *reuteri* caused a significant reduction (2.4-fold) in RANKL gene expression (*p*<0.05) and 1.8 fold increase in OPG expression. Analysis of the OPG:RANKL ratio revealed a significant elevation (2.4-fold) in the DSI + *L*. *reuteri* cohort when compared to the DSI mice (*p*<0.05). *L*. *reuteri* had no effect on BM IL-10 expression at 4 weeks. Together, these studies suggest that *L*. *reuteri* likely modulates genes responsible for bone remodeling in the BM. Whether these effects are consequences of intestinal changes or direct effect of *L*. *reuteri* secretory products will be the focus of future studies.

## Discussion

Recent studies have revealed a positive effect of probiotic supplementation on bone health in intact male mice and in estrogen-deficient female mice [23,24,26]. In contrast however, supplementation in intact female mice displayed no bone effect [26]. In the present study we reveal that dorsal skin incision (DSI) has long-lasting systemic effects on inflammatory status, and that treatment with the probiotic bacterium *L*. *reuteri* 6475 exerts a beneficial bone effect in female DSI mice in addition to modulating the expression of pro-inflammatory and pro-osteoclastogenic cytokines in the intestine and bone marrow.

Under normal physiological conditions inflammation is a controlled adaptive response by the body consequent to injury or infection, returning it to homeostasis. Dysregulation of this inflammatory process can lead to adverse pathology such as that seen in IBD, RA, estrogen deficiency and obesity [16–19,32]. Inflammatory cytokines can promote osteoclast bone resorption and bone loss. Previous studies investigating the effect of *L*. *reuteri* on bone health utilized intact male mice and the OVX model of estrogen deficiency; both models displaying significantly elevated markers of inflammation compared to intact female mice. In the present study, a model of non-pathological inflammation was required for *L*. *reuteri* to exhibit a bone effect in female mice.

Wound healing (surgery) induces a coordinated and controlled acute inflammatory response and was chosen over other models of inflammation such as low dose LPS- or DSS- induced inflammation, as these are closely related to pathological models of obesity and IBD respectively [33–36]. While inflammation associated with wound healing is generally considered localized to the site of insult, the present study revealed that at 8 weeks post-dorsal skin incision, general markers of systemic inflammation were still elevated: spleen, thymus and visceral fat weights were significantly increased. In addition, expression of pro-inflammatory genes, TNFα, IL-1β and IFNγ were elevated in the intestine. These markers indicated that the DSI mice, while visibly healed and showing no overt signs of illness or distress, were experiencing a low-grade systemic inflammation compared to the non-surgery controls.

In pathological states, such as RA or IBD, high levels of inflammation have an adverse effect on bone health. Pro- inflammatory and–osteoclastogenic cytokines such as, TNFα, IL-1β, IFNγ and RANKL, are increased while expression of the anti-osteoclastogenic cytokine OPG is decreased. This results in enhanced osteoclast formation and activity and suppressed osteoblast bone formation, leading to net bone loss [28,37–39]. The effect of low-grade inflammation on bone health however, is not as well defined. In adverse conditions such as obesity and diabetes, which are associated with a dysregulated chronic low grade inflammation, a lower BV/TV has been reported due to increased osteoclastogenesis and decreased osteoblast differentiation [20,25,40–42]. In the present study, no difference in trabecular BVF was observed between the non-surgery and the DSI mice. However, we did observe that DSI increased cortical bone parameters, including thickness, mineral content and calculated strength. MAR and serum osteocalcin levels were also elevated in the DSI mice, suggesting in this model that inflammation increased bone remodeling and that bone outcomes are site dependent. Specifically, surgery-induced changes were targeted to cortical bone where resorption and formation are not in balance and lead to structural changes. Osteocytes are known to play a critical role in the maintenance of homeostatic bone remodeling, through the production of RANKL [43,44] and could be involved in this process and contribute to site specific differences. We specifically examined bone marrow changes to assess the role of immune cells in DSI and *L*. *reuteri* responses. We identified that RANKL, OPG and IL-10 gene expression in DSI BM significantly increased along with the number of CD4^+^ T cells, another known producer of RANKL [1]. The results of these analyses support the notion that DSI increases bone remodeling that is in balance in trabecular bone but not in cortical bone. This effect is in contrast to what has been observed in obesity and diabetes, where RANKL is elevated and OPG and IL-10 expression reduced [42,45]. These observations suggest that the context of inflammation, controlled versus pathologic, has an important role in the outcome on bone health. Having identified that DSI induced a controlled systemic inflammatory response in female mice that had no detrimental effect on bone health, we demonstrate that supplementation with *L*. *reuteri* could produce the beneficial bone effect observed in male mice and OVX female mice.

In-line with previous reports for intact and diabetic male mice, *L*. *reuteri* had no effect on general body mass in either the non-surgery or DSI cohorts [25,26]. Consistent with previously published bone data supporting a requirement for inflammation in the response to probiotics, *L*. *reuteri* had an anabolic effect on bone only in the DSI cohort [23,26]. Also in line with this, 2-way ANOVA, used to examine if *L*. *reuteri* and surgery effects on mineral apposition rate were dependent or independent, determined that *L*. *reuteri* had no significant effect on MAR on its own. The exact mechanism through which *L*. *reuteri* increases bone density is currently not fully understood. However, in the present study *L*. *reuteri* modulated expression of BM RANKL, OPG and IL-10 gene expression, shifting the balance towards an anti-osteoclastogenic environment. Interestingly, the level of modulation was dependent on the presence of inflammation with the greatest changes observed in the DSI cohort, potentially due to the increased numbers of CD4^+^ T cells present in the BM. Studies have identified that probiotic bacteria can modulate T cell differentiation, up-regulating CD4^+^Foxp3^+^ Tregs while down-regulating Th1 and Th17 cytokine expression [46–48]. In the present study the numbers of CD4^+^Foxp3^+^ Tregs were not measured though the increase in expression of IL-10 and decreased RANKL gene expression suggests that *L*. *reuteri* is potentially modulating CD4^+^ T cell lineage selection. What remains to be determined however, is whether *L*. *reuteri* is modulating lineage selection within or before migration to the BM. While in the present study changes in BM gene expression were observed in the surgery cohort at 4 weeks a previous study from our laboratory reported no changes in bone density in sham operated mice at this time point [23]. This suggests that in female mice, under non-pathological low-grade inflammation, long-term supplementation with *L*. *reuteri* is required to increase bone density. The exact mechanisms of how intestinal microbiota affects bone health is still under investigation, though modulation of intestinal pro-inflammatory cytokine expression has been suggested to have an important role [26,49].

In the present study *L*. *reuteri* had regional and inflammation-dependent effects on intestinal cytokine gene expression. In the DSI cohort jejunal pro-inflammatory cytokine expression was reduced, supporting the observations in male mice by McCabe, Britton et al [26]. Interestingly however, an increase in colonic pro-inflammatory cytokine expression, notably TNF, was observed. Why *L*. *reuteri* would have region specific effects on cytokine expression in the intestine is not clear from this study, though a similar effect with commensal bacteria had been observed in gnotobiotic pigs [50]. A possible explanation for the regional difference in cytokine response is the difference in cellular make-up of the sections of the intestine; higher numbers and density of Peyer’s patches are found in the small intestine while equivalent M-cell containing macroscopic structures are found in the large intestine. Additionally 10- to 20-fold more intraepithelial lymphocytes can be isolated from the small intestine compared to the colon [51]. This suggests *L*. *reuteri* has differential effects and highlights a complex interplay between microbiota and host.

The *L*. *reuteri*-induced increase in intestinal pro-inflammatory cytokine expression is not without precedent. In a study by Pagnini *et al* [52] the probiotic cocktail VSL#3 stimulated ileal epithelial production of TNF. This increase in TNF was thought to protect against the onset of intestinal inflammation in the SAMP mouse model of Crohn’s disease-ileitis via local stimulation of the epithelial innate immune system, restoring epithelial barrier function. The effect of *L*. *reuteri* on epithelial barrier function in the current study cannot be conclusively determined. As with cytokine expression, region- and inflammation-dependent effects were apparent in epithelial tight junction protein mRNA expression. In the jejunum, under an inflammatory setting, *L*. *reuteri* increased mRNA expression of the tight junction protein occludin. In contrast, *L*. *reuteri* led to decreased occludin expression in the colon under non-inflammatory conditions. Whether these changes translate to changes in intestinal permeability however, remains to be investigated.

Comparison of the present study to the effects of *L*. *reuteri* on male mice and the estrogen-deficient OVX female mice raises a number of intriguing queries. Normal intact female mice display significantly lower intestinal inflammation compared to their male counterparts (unpublished data from our laboratory), while following estrogen deficiency inflammatory levels increase [32]. This suggests a minimal level of inflammation is required for *L*. *reuteri* to exert a beneficial bone effect. What this activating factor is however remains to be determined; whether it’s the presence of pro-inflammatory cytokines, certain activated immune cells in or around the intestine or some other hitherto unknown factor.

In summary, this study provides the first evidence that the probiotic bacteria *L*. *reuteri* requires a mild inflammatory status *in vivo* to provide a beneficial bone effect in female mice. We show that surgical dorsal skin incision induces a controlled systemic low -grade chronic inflammation that increases expression of pro-inflammatory cytokines in the intestine and the BM. We reveal that *L*. *reuteri* modulates expression of intestinal pro-inflammatory cytokines and tight junction protein expression in a regional- and inflammation-dependent manner and that *L*. *reuteri* can regulate BM gene expression of RANKL, OPG and IL-10. These data reveal the complex interaction between host and microbiota and the gut–bone axis.
